# New Electrodes and Battery for Electrolysis of Uterine Tumors

**Published:** 1878-12

**Authors:** 


New Electrodes and Battery for Electrolysis of Uterine Tumors. By E. Cutter, M. D., Cambridge, Mass.

A small pamphlet descriptive of new devices by Dr. Cutter, which overcome the difficulties ordinarily experienced in operating upon uterine fibroid tumors. The Dr. says, " in several instances, females who have given up this life, and awaited death, have been restored to the full performance of the active duties of healthy living."

The pamphlet is a candid confession of a want of complete knowledge of the conditions under which the softening and destruction of the adventitious tissue occurs, and thinks it an open question whether the galvanic current has anything to do with it, or in other words, whether the results would not be obtained by the punctures without the electricity." It is written in Dr. Cutter's usual modest but self-possessed syle. H.

